# Photocatalytic Reduction of Methylene Blue by Surface-Engineered Recombinant *Escherichia coli* as a Whole-Cell Biocatalyst

**DOI:** 10.3390/bioengineering10121389

**Published:** 2023-12-04

**Authors:** Ashokkumar Kumaravel, Vidhya Selvamani, Soon Ho Hong

**Affiliations:** Department of Chemical Engineering, University of Ulsan, 93 Daehak-ro, Nam-gu, Ulsan 44610, Republic of Korea; bioashok00793@gmail.com (A.K.); svidhudolt@gmail.com (V.S.)

**Keywords:** methylene blue, photo-catalysis, *Escherichia coli*, adsorption, nanoparticle

## Abstract

A novel *Escherichia coli* strain, created by engineering its cell surface with a cobalt-binding peptide CP1, was investigated in this study. The recombinant strain, pBAD30-YiaT-CP1, was structurally modeled to determine its cobalt-binding affinity. Furthermore, the effectiveness and specificity of pBAD30-CP1 in adsorbing and extracting cobalt from artificial wastewater polluted with the metal were investigated. The modified cells were subjected to cobalt concentrations (0.25 mM to 1 mM) and pH levels (pH 3, 5, 7, and 9). When exposed to a pH of 7 and a cobalt concentration of 1 mM, the pBAD30-CP1 strain had the best cobalt recovery efficiency, measuring 1468 mol/g DCW (Dry Cell Weight). Furthermore, pBAD30-CP1 had a higher affinity for cobalt than nickel and manganese. Field Emission Scanning Electron Microscopy (FE-SEM), Transmission Electron Microscopy (TEM), and Energy-Dispersive X-ray Spectroscopy (EDS) were used to examine the physiochemical parameters of the recombinant cells after cobalt adsorption. These approaches revealed the presence of cobalt in a bound state on the cell surface in the form of nanoparticles. In addition, the cobalt-binding recombinant strains were used in the photocatalytic reduction of methylene blue, which resulted in a 59.52% drop in the observed percentage. This study shows that modified *E. coli* strains have the potential for efficient cobalt recovery and application in environmental remediation operations.

## 1. Introduction

Being flexible and adaptable, metals are utilized throughout various fields, including electronics, medicine, construction, jewelry, agriculture, food, furniture, security systems, machinery, and automotive industries. Despite their extensive utilization, the long-term exploitation of natural resources has resulted in exhaustion. Fortunately, the recyclability of metals provides a valuable opportunity to recover and conserve resources. Several heavy metals are required for biological activity, but their safety must be monitored [1]. Heavy metals have two essential characteristics: harmful effects on low-dose living creatures and bio-accumulative properties. Plants and bottom sediments rich in heavy metals become poisonous over time, endangering all living species [2]. As a result, their presence in aqueous water streams has become a significant issue to be addressed. The potential toxicity is based on a range of parameters, including dosage, exposure route, chemical species, and the aging, sex, ethnicity, and overall health of those exposed [3]. Cobalt, a very adaptable metal, is abundantly present in the Earth’s crust and has been historically esteemed for its characteristic blue coloration, which has made it a popular choice for use in jewelry and paints [4]. Co limitations for farm water and livestock wastewater are 0.05 mg/L and 1 mg/L (Environmental Bureau of Investigation, Canadian Water Quality Guidelines). Co-metal was the subject of a Harmonized Classification and Labelling (CLH) dossier presented by the Netherlands in 2016, which included information on the metal’s possible carcinogenic, mutagenic, and reproductive toxicity [2]. The high γ-energies (1.17 and 1.33 MeV) and long half-life (5.27 years) of ^60^Co have made it an essential factor in person-sievert budgeting, even though the total cobalt concentration (^59^Co + ^60^Co) is relatively low compared to Fe, Ni, and Cr when the oxide film is dissolved in a chemical formulation [5]. Recently, cobalt has been increasingly employed in many applications, such as alloys, batteries, and electroplating. Rechargeable batteries constitute approximately 25% of the worldwide demand for cobalt, as cobalt was a substantial constituent within lithium-ion batteries (LIBs) in 2007 [6], alongside lithium, nickel, organics, and plastics [7,8]. The rapid growth of the electronics sector has led to an increase in the disposal of electronic equipment, resulting in a significant rise in electronic waste (EW). This issue is particularly concerning due to the presence of important metals such as cobalt and lithium within electronic trash. The utilization of cobalt in lithium-ion batteries (LIBs) has experienced a significant surge, hence requiring the retrieval of cobalt from spent electrode waste (EW) to alleviate its escalating expenses [9]. The increasing need for portable electronic devices such as mobile phones, laptops, computers, and cameras intensifies this difficulty [7,10,11].

Regrettably, the infiltration of metals from discarded electronic waste (EW) into industrial wastewater results in their subsequent release into the environment. Especially when cobalt enters the environment, it cannot be destroyed. It may react with other particles or absorb soil particles or water sediments. The process of recycling metals has the potential to mitigate the burden on natural resources, decrease the expenses related to battery manufacture, and improve the environmental concerns linked with these activities [12]. Examining cobalt’s environmental occurrence is of utmost importance because of its potential health hazards when present in high quantities, which may result in adverse effects such as hypertension [13], paralysis and skeletal abnormalities [14], and gastrointestinal disturbances [14]. Furthermore, cobalt is classified as a perilous radioactive substance possessing a protracted half-life [15]. Hence, there exists an urgent requirement for the cleanup of cobalt. Numerous research studies have been conducted to examine cobalt extraction from spent lithium-ion batteries (LIBs) using different methodologies. Adsorption [16], ion exchange [17], nanofiltration [18], polyurethane foams [19], cobalt-imprinted polymer [20], and Co selective modified chitosan have all been studied in the laboratory to extract Co [21].

However, these approaches frequently demonstrate limited efficacy when applied to lower amounts of cobalt [22]. Recently, there has been an increasing inclination towards harnessing the potential of microorganisms in the domain of metal recovery, with a focus on utilizing bioleaching and biosorption techniques [23]. Biosorption, which entails the adsorption of metals onto the surface of microorganisms, offers a potentially viable alternative for recovering metals in environmental contexts [21,24,25]. The utilization of Cell Surface Display (CSD) to enhance the efficacy of biosorption by employing short metal-binding peptides presents a targeted strategy for retrieving metals [26].

**Figure 1:**
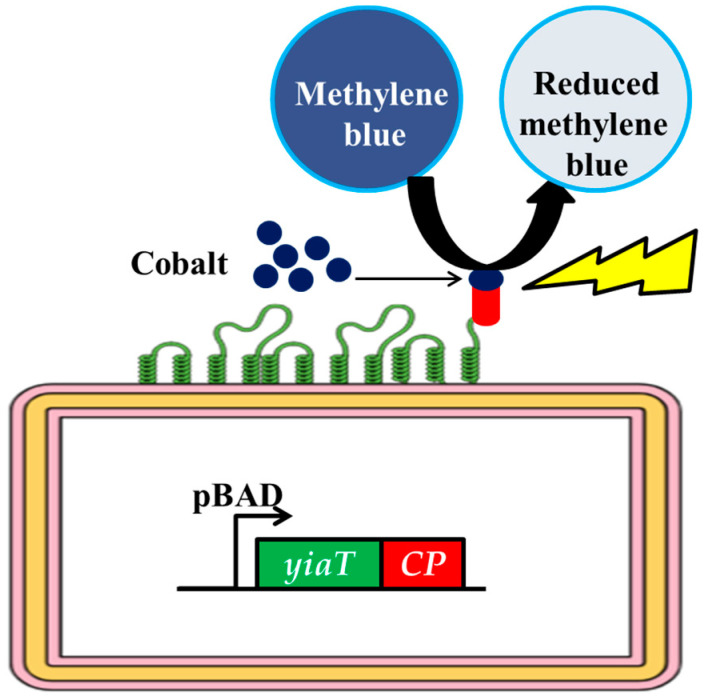
Cell surface display of cobalt-binding peptide as whole-cell biocatalyst towards reduction of methylene blue.

This study investigated cobalt-binding peptide (CP1) cellular surface display (CSD) on *Escherichia coli*, employing YiaT as the anchoring protein. The system’s efficiency for cobalt recovery was evaluated in artificially polluted wastewater (AWW). To visually represent and cartographically analyze the distribution of cobalt on the cellular membrane of genetically modified *Escherichia coli*, we utilized Scanning Electron Microscopy (SEM), Transmission Electron Microscopy (TEM), and Energy-Dispersive X-ray Spectroscopy (EDX). The cobalt attached to the cell’s surface was then utilized in the photocatalytic reduction of methylene blue [27,28] (Figure 1). This study addresses the requirement for environmentally sustainable methods to treat and remediate pollutants from wastewater.

## 2. Materials and Methods

### 2.1. Bacterial Strains and Growth Conditions

The bacterial strains used in this study are listed in Table 1. The strains were grown in LB medium (10 g/L bacto-tryptone, 5 g/L bacto-yeast extract, and 5 g/L NaCl) with 100 mg/L ampicillin at 37 °C with vigorous shaking at 250 rpm.

### 2.2. Recombinant Plasmid Construction

The cobalt-binding peptide gene was successfully incorporated into the C-terminus of the shortened yiaT protein, precisely at the 5th loop, which consists of 696 base pairs. The amplification of the fusion construct was achieved by employing the polymerase chain reaction (PCR) technique, utilizing the MJ Mini Personal Thermal Cycler (Bio-Rad Laboratories, Hercules, CA, USA) and the Expand High Fidelity PCR equipment (Roche Molecular Biochemicals, Mannheim, Germany). The peptide sequence CP1, which serves as an integrated cobalt-binding peptide, is represented by the following nucleotide sequence: CATTATCCGACCCTGCCGCTGGGCAGCAGCACCAC. The design of these sequences was customized to conform to the codon usage patterns. The precise primers utilized in this method are documented in Table 2. The primers with YiaT-CP1 were inserted into the ampicillin-resistant, arabinose-inducible promoter containing plasmid pBAD30 using *SacI* and *KpnI* restriction enzymes, forming pBAD30CP1. Arabinose was employed to induce the production of the fusion protein YiaT-CP1. Subsequently, the plasmids harboring these constructions were inserted into *Escherichia coli*’s Top 10 strains for additional research.

### 2.3. SDS Page Analysis

The *E. coli* strain expressing the pBAD30 YiaT-CP1 protein was cultivated for 12 h in LB medium at 37 °C. Subsequently, the cultures were diluted 100-fold using a fresh LB medium. Following an optical density at 600 nm (OD600) of 0.5, arabinose was introduced into the culture medium at concentrations ranging from 0 to 1% and at different temperatures from 25 °C to 30 °C for 5 h to determine the optimal expression. The recombinant strains were collected using centrifugation at a speed of 13,000× *g* for 10 min. Subsequently, they were incubated with B-7M urea buffer (7 M Urea, 0.1 M NaH_2_PO_4_, 0.01 M Tris, pH 8) at ambient temperature for 30 min while agitated. The cellular samples underwent centrifugation at a speed of 5000× *g* to eliminate any residual cellular debris. The cell pellet was subjected to separation of the outer membrane fractions by adding a 10 mM Tris-HCl solution at pH 7.5. The suspended cells were then stored at 4 °C for 12 h. The membrane fractions that were elevated were subjected to analysis using a 12% (*w*/*v*) sodium dodecyl sulfate-polyacrylamide gel electrophoresis (SDS-PAGE) technique [29]. The fractions were stained with Coomassie brilliant blue R-250 (Bio-Rad Laboratories, Hercules, CA, USA).

### 2.4. Molecular Modeling Studies

The computational analysis was conducted to investigate the cobalt-binding peptide CP1 exhibited by YiaT. The transmembrane protein model was analyzed using PRED-TMBB, a Hidden Markov tool to predict and differentiate beta-barrel outer membrane proteins [30]. The peptide YiaT-CP1’s three-dimensional structure was generated using the I-TASSER server and Swiss model online software tools [31]. The Ramachandran plot for the CSD with its YiaT-CP1 peptides was analyzed through the Swiss model and I-TASSER [32]. Additionally, the predictive capability of MIB [33,34] was employed to determine the affinity of peptides for cobalt binding.

### 2.5. Cobalt Recovery Analysis

The recombinant strain pBAD30-CP1 was cultured at 37 °C in LB medium supplemented with 100 mg/L of ampicillin and incubated for 12 h. Then, the culture was diluted 100-fold in freshly made LB media and cultivated until the optical density at 600 nm reached a value of 0.5. Subsequently, the bacterial cells were subjected to a 0.05% arabinose solution and incubated at 30 °C for 6 h. Subsequently, the bacterial strains underwent centrifugation at 8000× *g* for 10 min, 4 °C and were treated with cobalt metal chloride solutions with varying concentrations ranging from 0.25 mM to 1 mM at different pH values from pH (3, 5, 7, 9) and the incubation period lasted 30 min at a controlled temperature of 30 °C, accompanied by agitation at 250 rpm.

To remove the cobalt ions that had been adsorbed on the surface of the cells, the bacterial strains underwent a washing technique involving a 0.85% (*w*/*v*) NaCl solution, followed by treatment with 1 mM EDTA for 30 min in ice. The peptides’ adsorption capability was assessed by employing anticipated pollutants. The adsorption performance of the most effective recombinant strain was analyzed using an Inductively Coupled Plasma Optical Emission Spectrometer (ICP-OES), with *E. coli* TOP10 utilized as a reference. The recombinant strains’ selectivity was assessed throughout a range of concentrations (0.25 mM to 1 mM) for cobalt, nickel, and manganese with different pH levels (3, 5, 7, 9). The analysis was conducted via inductively coupled plasma optical emission spectroscopy (ICP-OES).

### 2.6. FE-SEM, TEM, and EDS Analysis

The cobalt-adsorbed strains were visualized using FE-SEM (JEOL JSM-6500F) and TEM. After adsorption, the recombinant *E. coli* cells were washed with 0.85% NaCl (pH 5.8) to remove the unbound cobalt on the cell surface. After washing, the cells were fixed and incubated at 4 °C for 14 h using 2.5% glutaraldehyde. The fixed cells were washed with PBS, mounted on ultra-flat silicon wafers, and dried to visualize them on an FE-SEM. The cobalt-adsorbed recombinant *E. coli* was fixed with the above procedure and coated on copper grids to visualize through TEM. Further, EDS analysis mapped the metal elements on the cell surface.

### 2.7. Photocatalytic Properties of Cobalt Nanoparticle

The degradation of methylene blue dye evaluated the photocatalytic efficacy of cobalt nanoparticles. The cell was combined with 100 mL of a methylene blue solution containing a concentration of 10 ppm to get the desired outcome. The solution was maintained in a dark environment for 30 min to achieve adsorption-desorption equilibrium. Subsequently, it was exposed to solar light provided by a 150 W Xenon lamp equipped with a cutoff filter. The samples were collected at consistent time intervals of (0, 10, 30, 60, and 90 min). The samples underwent filtration using a Millipore filter and were then examined using a UV-visible spectrophotometer set at a wavelength of 664 nm. To assess the efficacy of photocatalysis, a control experiment was conducted under comparable conditions. The experiment on dye degradation was conducted in triplicate under ambient conditions. The estimation of dye degradation % was performed by implementing the equation presented in Equation (1).

(1) $$\mathrm{Degradation}\;\mathrm{percentage}=\left[\frac{\left(C_{i}-C_{f}\right)}{C_{i}}\right]100$$

where $C_{i},\;$ initial concentration of methylene is blue and $C_{f}$ is dye concentration after degradation.

## 3. Results and Discussion

### 3.1. Molecular Modeling and Docking

**Figure 2:**
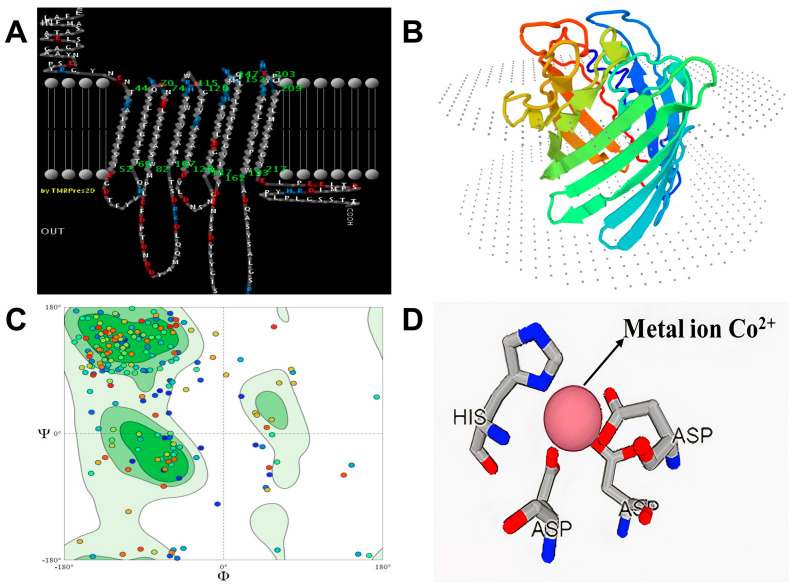
Molecular modelling and binding potential of YiaT-CP1. (**A**) 2D modelling of transmembrane protein by PRED-TMBB. (**B**) 3D structure of YiaT-CP1 generated using the I-TASSER server and Swiss model expasy. (**C**) The Ramachandran plot for YiaT-CP1 peptides by Swiss model and I-TASSER. (**D**) Pictorial representation for affinity of CP1 for cobalt ions.

Utilizing the Yiat-CP1 cobalt-binding peptide sequence allows the online tool PRED-TMBB to estimate a protein’s Cell Surface Display (CSD). The prediction score generated by the program was 2.919, which is nearly close to the predetermined threshold of 2.965. A minimal difference between the prediction score and the predetermined threshold signifies a strong probability that the protein is an outer membrane protein (Figure 2A). Therefore, based on the criterion, the protein YiaT-CP1 can be categorized as an outer membrane protein. The peptides YiaT-CP1 were subjected to molecular structure modeling using the C-I Tasser and Swiss Model Expassy online tools. The modeling results indicated that the peptides adopt a beta-barrel structure, confirming their classification as outer membrane proteins (Figure 2B). The Ramachandran plot of the YiaT-CP1 peptide was examined using the Swiss model. The Ramachandran plot is a graphical representation that visualizes the backbone dihedral angles to the amino acid residues inside a protein structure. The favored regions were calculated using a dataset of 12,521 non-redundant experimental structures obtained from PISCES. These structures were selected based on a pairwise sequence identity criterion of 30% and an X-ray resolution cut-off of 2.5 Å. Subsequently, histograms with a binning resolution of four degrees were employed to tally the occurrences of Φ Phi (C-N-CA-C) and N-CA-C-N (Psi Ψ) across all the shown categories. The determination of contour lines is based on quantifying observed Φ/Ψ pairings (Figure 2C). The colored dots correspond to distinct locations in the conformational space of the protein structure’s backbone dihedral angles φ (phi) and ψ (psi). The colors are usually designated to emphasize the extent to which sterically permitted or prohibited conformations are present, as determined by observable protein structures. Favored Regions: These regions correspond to dihedral angles within the observed ranges in well-folded protein structures, as determined by empirical studies. Dots in these places are frequently tinted with hues of green or blue. Allowed Regions: Certain combinations of dihedral angles are less frequent but still acceptable; dots in these places could be colored yellow. Disallowed regions: These are areas where the dihedral angles are infrequently observed because of steric collisions or other unfavorable interactions; dots in these places are frequently tinted with various colors of red. Little dots inside the green region of a Ramachandran plot signify that the dihedral angles φ (phi) and ψ (psi) for particular residues are located within the preferred or permissible regions, as determined by empirical observations of adequately folded protein structures. The molecular structure of peptide binding to the cobalt was illustrated by using the MIB server, which will also determine the binding potential of the peptides (Figure 2D).

### 3.2. Optimization of Expression Conditions

**Figure 3:**
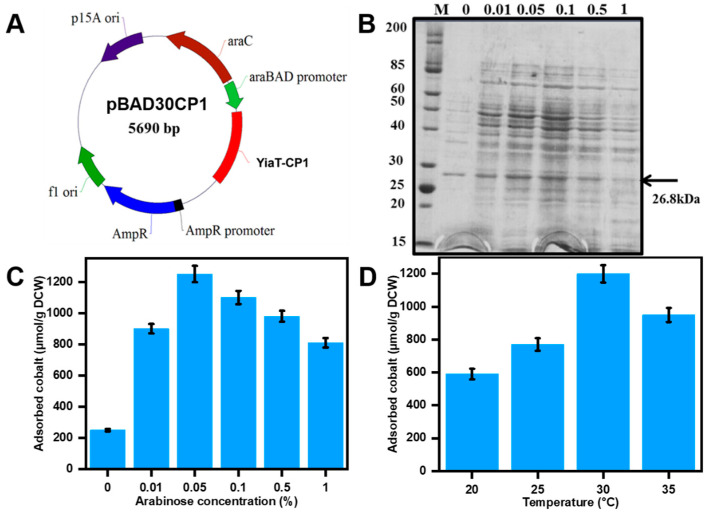
Construction of cobalt-binding peptide fused with YiaT at pBAD30 and optimization of its expression conditions. (**A**) Plasmid construction of cobalt-binding peptide CP1 fused with YiaT at pBAD30 (**B**) SDS-PAGE analysis of recombinant protein *E. coli* (YiaT-CP1) (26.8 kDa). (**C**) The effect of arabinose concentration towards cobalt recovery on *E. coli* (YiaT-CP1) with 1 mM CoCl_2_. (**D**) The effect of temperature towards cobalt recovery on *E. coli* (YiaT-CP1) with 1 mM CoCl_2_.

Recombinant *E. coli* was used in the context of cobalt recovery. This was achieved by expressing the cobalt-binding peptide CP1 on the cell membrane’s outer surface, facilitated by utilizing YiaT as an anchoring motif. The shortened YiaT gene was merged with CBP at 696 base pairs to implement this method. The plasmid utilized in this study is pBADCP1, which is under the control of the arabinose promoter, as depicted in Figure 3A.

In general, the expression of a heterologous protein in *E*. *coli* results in metabolic instability. Consequently, the expression of proteins, cellular proliferation, and durability of the recombinant plasmid are all diminished [23]. Therefore, identifying optimal growth and expression conditions is a crucial factor. The impact of varying arabinose concentrations and culture temperatures was assessed. The optimization of YiaT-CP1 peptide expression involves adjusting the arabinose concentration within the range of 0.05–1.0% and the temperature within 20–35 °C. The expression of the recombinant peptide, *E. coli* pBADCP1, was subsequently assessed through SDS-PAGE analysis (Figure 2B). The most favorable expression was observed at an arabinose concentration of 0.05% with the adsorbed cobalt concentration of 1250 µmol/g DCW. The maximum cobalt adsorption was recorded at 30 °C with a cobalt chloride concentration of 1 mM, yielding 1200 µmol/g DCW.

### 3.3. Cobalt Bio-Adsorption Studies

**Figure 4:**
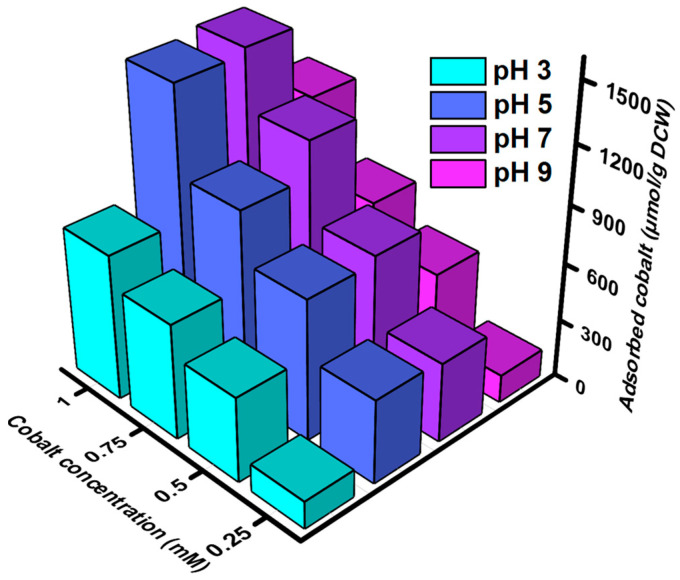
The adsorption intensity of cobalt-binding peptide *E. coli* (pBAD30-CP1) at different pH and concentrations. The datasets were obtained by averaging triplets with a deviation of ±5%.

Adsorption of cobalt by the peptides pBAD30-CP3 was evaluated by culturing the recombinant *E. coli* in an LB medium supplemented with ampicillin. The peptides were overexpressed by adding arabinose at 30 °C for 5 h. The adsorption characteristics of the CP1 cobalt-binding peptide were examined under varying pH conditions and induced concentrations. The investigation focused on assessing the recovery rates of the peptide, quantified in (μmol/g DCW (Dry Cell Weight)), across different experimental circumstances (Figure 4). At a pH of 3, the peptide demonstrated varying adsorption intensities at induced concentrations of 0.25 mM, 0.5 mM, 0.75 mM, and 1 mM. These concentrations corresponded to 143 µmol/g DCW, 441 µmol/g DCW, 600 µmol/g DCW, and 757 μmol/g DCW recovery rates. Upon adjusting the pH to 5, it was seen that the recovery rates experienced a significant rise. Specifically, the recovery rates for the corresponding concentrations of cobalt reached 435 µmol/g DCW, 733 µmol/g DCW, 987 µmol/g DCW, and 1437 μmol/g DCW. At a pH of 7, the peptide exhibited adsorption behavior resulting in recovery rates of 413 µmol/g DCW, 767 µmol/g DCW, 1168 µmol/g DCW, and 1468 μmol/g DCW at induced concentrations of 0.25 mM, 0.5 mM, 0.75 mM, and 1 mM, respectively. In the case of cobalt, it was shown that the recovery rates at a pH of 9 were 145 µmol/g DCW, 489 µmol/g DCW, 691 µmol/g DCW, and 1085 μmol/g DCW at the corresponding quantities. Hence, while comparing the various pH levels, it becomes apparent that pH 5 and pH 7 exhibit markedly superior recovery rates compared to pH 3 and pH 9. The selection of pH level is crucial to maximizing the efficacy of this peptide for cobalt binding. pH 5 and pH 7 are considered advantageous choices, but lower efficiency is exhibited at the pH values of 3 and 4.

The selectivity of pBAD30-CP1 towards cobalt was evaluated via an experimental investigation utilizing an NCM chloride solution (Nickel, Cobalt, Manganese). The experimental procedure entailed exposing the recombinant strains pBAD30-CP1 to different concentrations (0.25–1 mM) of artificially made-up wastewater (AWW), which contain cobalt (Co), nickel (Ni), and manganese (Mn) chloride solutions in distilled water.

**Figure 5:**
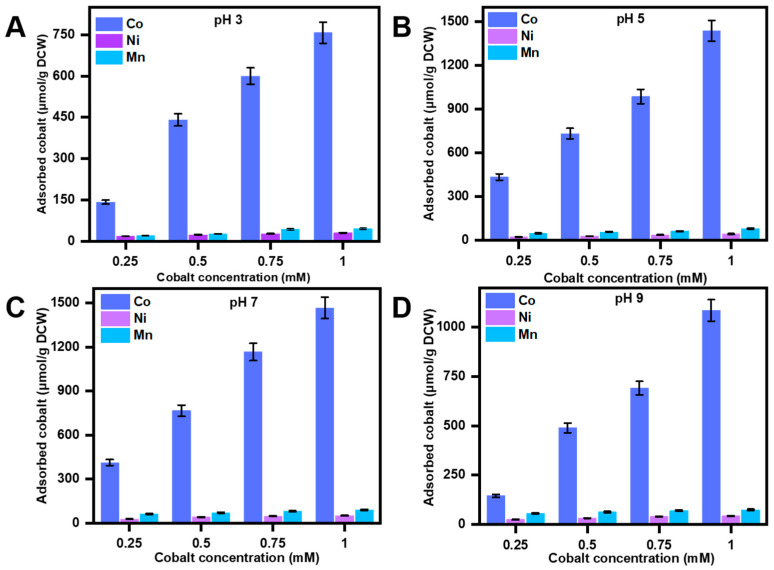
The selectivity of cobalt-binding peptide *E. coli* (pBAD30-CP1) NCM chloride solution with different pH. (**A**) pH 3, (**B**) pH 5, (**C**) pH 7, (**D**) pH 9. The datasets were obtained by averaging triplets with a deviation of ±5%.

The adsorption of pBAD30-CP1 at pH 3 with 1 mM NCM-induced concentration resulted in a recovery of 757 µmol/g DCW, 31 µmol/g DCW, and 46 µmol/g DCW of Co, Ni, and Mn, respectively (Figure 5A). Upon adjusting the pH to 5, it was seen that the recovery rates significantly rose under the same conditions of 1 mM concentration. This resulted in cobalt, nickel, and manganese exhibiting 1437 µmol/g DCW, 45 µmol/g DCW, and 81 µmol/g DCW recovery rates, respectively (Figure 5B). The maximum recovery for all three metals, with respective values of 1468 µmol/g DCW, 52 µmol/g DCW, and 91 µmol/g DCW, was observed at a pH of 7 and an induced concentration of 1 mM (Figure 5C). In contrast, when the pH was adjusted to 9, a concentration of 1 mM was produced. The recovery values for cobalt, nickel, and manganese were measured as 1085 µmol/g DCW, 43 µmol/g DCW, and 74 µmol/g DCW, respectively (Figure 5D). The specificity of pBAD30-CP1 was maintained at all pH levels, and only a negligible quantity of nickel and manganese was adsorbed by the peptides. Our recombinant peptide pBAD30-CP1 demonstrates a remarkable specificity towards cobalt, exhibiting 25.23 and 14.66 times higher affinity for cobalt as compared to nickel and manganese respectively, at pH 7 and 1 mM NCM chloride solution.

The influence of the pH of the medium on the recovery efficiency of cobalt, nickel, and manganese by pBAD30-CP1 becomes apparent. The optimal pH for these metals’ recovery is 7, with a pH of 5 also exhibiting favorable recovery rates. On the contrary, a pH value of 3 leads to diminished recovery effectiveness, whereas a pH of 9 exhibits somewhat lesser efficiency but remains feasible for retrieving these metallic elements.

### 3.4. Physiochemical Characterization of Bio-Adsorbed Cobalt

#### FE-SEM, TEM, and EDS Analysis

**Figure 6:**
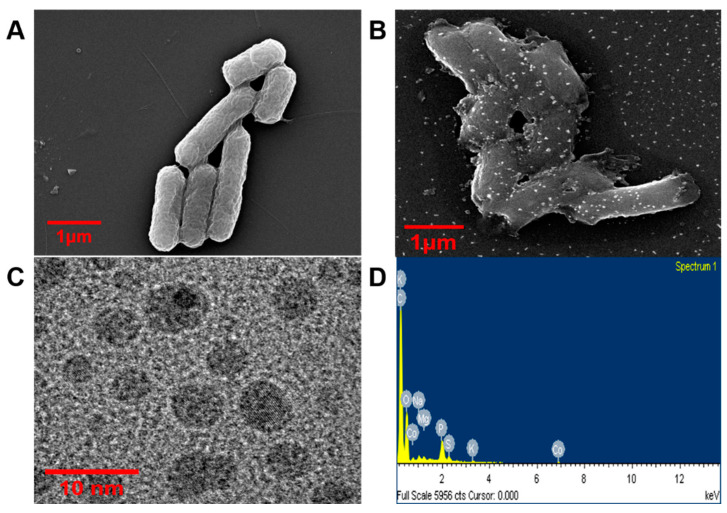
Morphological characterization of recombinant strain *E. coli* (pBAD30-CP1) after cobalt adsorption. (**A**) FE-SEM of control *E. coli*. (**B**) FE-SEM image of cobalt adsorbed *E. coli* (pBAD30-CP1) strain, (**C**) TEM images of nano-sized cobalt particles adsorbed by CP1 displayed recombinant *E. coli*., (**D**) EDS spectrum of cobalt adsorbed recombinant *E. coli* (pBAD30-CP1).

The current investigation entailed the examination of recombinant strain pBAD30-CF1 through utilizing FE-SEM, TEM, and EDS methodologies after cobalt absorption. The main aim of this investigation was to visually represent the adsorbed metallic element and ascertain its structural features. After adsorption, the cells were subjected to a washing and lyophilization procedure using an FE-SEM to facilitate their analysis. The application of FE-SEM analysis demonstrated the presence of nanoparticles on the surface of recombinant pBAD30-CF1 following the adsorption of cobalt at a concentration of 1 mM cobalt solution (Figure 6B). Based on the results obtained from the TEM examination, it can be deduced that the cobalt nanoparticles, characterized by a size distribution ranging from 5 to 8 nm, were primarily attached to the cellular membrane (Figure 6C). The detection of cobalt nanoparticles in the strains was achieved by identifying spectral peaks, specifically Co Kα1 and Co Kβ1, which were observed at energy levels of 6.931 keV and 7.649 keV, respectively, utilizing EDS (Figure 6D). The control strain of *Escherichia coli*, TOP10, did not demonstrate the occurrence of nanoparticle production on its cell wall (Figure 6A). In contrast, the recombinant *Escherichia coli* strain exhibited the existence of vivid cobalt salt nanoparticles on the exterior of its cellular membrane. The results obtained from the experiment on cobalt adsorption indicate that the cell wall surface of recombinant *E. coli* harbors a substantial number of binding sites specifically for cobalt ions. The ionic species on the cellular membrane can engage in chemical reactions, creating inorganic compounds that demonstrate precipitation from the solution in the immediate vicinity. These specific places can accumulate ions within a limited space, enhance the extent of coverage on the surface, and promote the clustering of ions, ultimately forming solid nanoparticles on the cellular membrane.

### 3.5. Photo-Catalytic Degradation Activity with Methylene Blue

The process of the reaction mechanism commences with the excitation of electrons from the valence band (VB) towards the conduction band (CB), thereby creating a vacancy in the VB. The VB-generated perforations concomitantly engage with the surface-bound water molecule or hydroxyl ion, forming hydroxyl radicals (^●^OH). Upon immediate contact, the electrons in the conduction band situated on the surface of the nanoparticles undergo a reaction with the dissolved oxygen molecule, forming superoxide radicals (^●^O_2_^−^). The electron-hole pair recombination was avoided through the involvement of ^●^O_2_^−^ in the oxidation process, thereby preserving electron neutrality within the photocatalyst. Hydrogen peroxide (H_2_O_2_) production occurred through hydroperoxyl radicals’ protonation, followed by the dissociation of H_2_O_2_, which subsequently generated ^●^OH radicals [35]. Cobalt nanoparticles generate a potent oxidizing agent in the form of reactive species that proceeds to initiate an attack on the dye molecules [36,37]. The reactive species attack the organic dye molecules, leading to their mineralization and subsequent water molecules and carbon dioxide formation. The reactive species primarily govern the photodegradation mechanism of methylene blue dye ^●^OH and ^●^O_2_^−^ [38,39].

**Figure 7:**
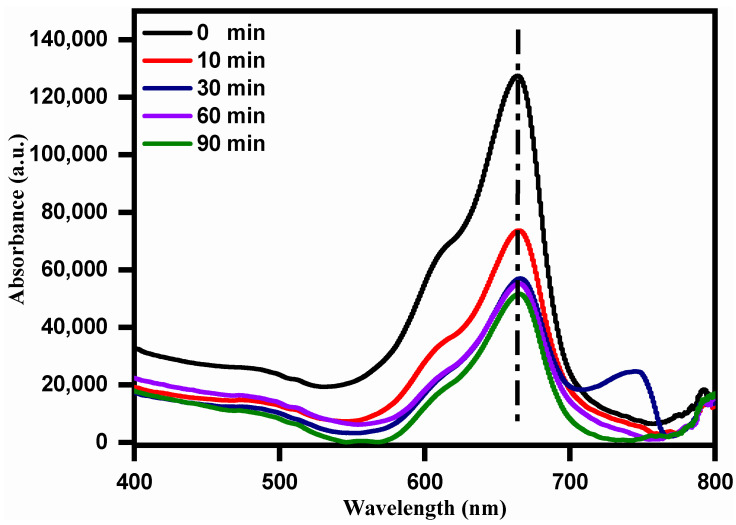
UV absorption spectra of methylene blue photocatalytic degradation using cobalt nanoparticles biosynthesized by surface displayed *E. coli* (pBAD30-CP1).

Photocatalytic degradation of methylene blue (MB) dye was studied using recombinant *E. coli* displayed with CP1 after cobalt adsorption. In the UV-Vis spectrum, MB shows a maximum of 664 nm. The MB solution was mixed with cell surface-bound cobalt particles and exposed to solar light. The sample was retrieved at regular time intervals and analyzed UV-Vis spectroscopy after filtration. It could be observed that the peak at 664 nm was reduced with an increase in time. This further confirms the photocatalytic degradation of MB by cobalt nanoparticles. The photocatalytic activity towards methylene blue degradation was 59.52% after 90 min of light irradiation (Figure 7). These studies, with our findings, show the novelty of the whole-cell cobalt nanoparticles with significant photocatalytic degradation of dyes.

## 4. Conclusions

Cobalt, a notable metal pollutant within electrical waste (EW), can be a valuable secondary raw material. This study has demonstrated the feasibility of developing a highly efficient cobalt recovery system utilizing the Cell Surface Display (CSD) technique, employing synthetic cobalt-binding peptides. Specifically, the peptide CP1 was successfully presented on the cell surface using YiaT as an anchoring motif. The results indicate substantial cobalt recovery capabilities, with recorded values of 1437 μmol/g DCW and 1468 μmol/g DCW when exposed to 1 mM concentrations of cobalt chloride at pH 5 and pH 7, respectively. Towards specificity, our recombinant peptide pBAD30-CP1 exhibits 25.23 and 14.66 times higher specificity towards cobalt than nickel and manganese. Additionally, Scanning Electron Microscopy (SEM) and Transmission Electron Microscopy (TEM) were employed to elucidate the morphological features of cobalt-bound recombinant cells. Furthermore, Energy-Dispersive X-ray Analysis (EDX) confirmed the presence of cobalt, revealing that cobalt particles adhering to the cell surface are in the form of nano-sized spheres. This research underscores the promising potential of CSD of synthetic cobalt-binding peptides for the efficient recovery of cobalt from environmental sources, contributing to valuable recycling efforts in electronic waste management. Further recombinant cells with adsorbed cobalt on the surface were used as a whole-cell biocatalyst for the photocatalytic degradation of methylene blue, resulting in an observed degradation efficiency of 59.52%.

## Figures and Tables

**Table 1:** List of bacterial strains and plasmids used in this study.

Strain/Plasmid	Relevant Genotype/Property	Source
*E. coli* Strains		
TOP10	F-mcrA Δ(mrr-hsdRMS-mcrBC) φ80lacZΔM15 ΔlacX74 nupG recA1 araD139 Δ(ara-leu)7697 galE15 galK16 rpsL(Str ^R^) endA1 λ^−^	Stratagene
**Plasmids**		
pBAD30	Amp ^R^	NEB ^a^
pBADCP1	pBAD30 containing YiaT-CP1	This work

^R^—Resistance, ^a^ New England Biolabs, Beverly, MA, USA.

**Table 2:** Primers used in this work.

Name	Sequence (5′ to 3′)
C_F	GAGCTCATGTTAATTAATCGCAATATTGTGGCGTTATTTG
CP_3__R	GGTACCGGTGGTGCTGCTGCCCAGCGGCAGGGTCGGATAATGACGATCAATCATCGGGCTGTCGGTAAT

## Data Availability

Raw data supporting the conclusions of this article will be made available by the authors upon request.
